# Linear accelerator utilization: Concept and tool to aid the scheduling of patients for radiotherapy

**DOI:** 10.1016/j.tipsro.2021.09.001

**Published:** 2021-09-30

**Authors:** Jesper Lindberg, Thomas Björk-Eriksson, Caroline E. Olsson

**Affiliations:** a Medical Radiation Sciences, Institute of Clinical Sciences, Sahlgrenska Academy, University of Gothenburg, 413 45 Gothenburg, Sweden; b Department of Medical Physics and Biomedical Engineering, Sahlgrenska University Hospital, 413 45 Gothenburg, Sweden; c Regional Cancer Centre West, Western Sweden Healthcare Region, 413 45 Gothenburg, Sweden; d Department of Oncology, Institute of Clinical Sciences, Sahlgrenska Academy, University of Gothenburg, 413 45 Gothenburg, Sweden

**Keywords:** Radiotherapy, Scheduling, Capacity, Utilization, Tool, **LUR**, Linac utilization rate, OIS, Oncology information system

## Abstract

**Background and purpose:**

Resources in radiotherapy (RT) need to be used effectively to meet the current clinical demand. The aim of this data-driven study is to identify temporal trends in the scheduling of patients for RT and to develop a tool for a visual overview of future scheduling levels.

**Material and methods:**

Scheduling data at an eight-linac modern RT department in Sweden were collected twice daily for planned and observed linac use in 2018–2020. Information was retrieved each day for the present (Day 0) and the forthcoming 100 weekdays with total linac utilization rates (LURs) calculated for two activity categories: *treatment* and *non-treatment*. An in-house tool based on the LUR concept, database queries from the oncology information system (OIS)/automatic calculations was developed and evaluated by RT managers and scheduling staff (n = 10).

**Results:**

Overall median LURs were 87%/89% (planned/observed; p < 0.01) with more frequent and larger daily increase for non-treatment activities compared with treatment activities. LUR increased with shorter planning horizons and reached 100% for fully-operating linacs ≈3 weeks before Day 0. The tool was reported by 88% to ease the work and to contribute towards an even scheduling of patients (responses: 8/10).

**Conclusion:**

Alterations from a planned RT schedule occurs frequently. Having a tool that helps to reduce the abundance of booking information into clinically relevant overviews promise to increase the understanding of present and future scheduling levels. Our proposed concept and tool suggest that this is a feasible approach to schedule patients for RT more evenly.

## Highlights

• Linacs were fully booked already three weeks before a majority of investigated days.
• Non-treatment events were scheduled ≤ 11% for the investigated time period.
• Planned booking scenarios deviated from real scenarios for most investigated days.
• Largest changes were primarily related to linac downtime/maintenance issues.
• Tool visualizing linac utilization may aid staff to achieve an even RT workflow.

## Introduction

Cancer is increasing globally and therefore the need for radiotherapy (RT) [1]. About 50% of the European cancer patients are recommended RT, but there is a large gap between actual and desirable use of RT [2]. Scheduling of RT is challenging for many reasons. Time slots for linear accelerators (linacs) need to accommodate variable patient inflows, different treatment objectives/complexities, and different overall treatment time [3]. Overall treatment capacity is restricted by departmental opening hours and the number of available linacs, and depends on the presence of a multidisciplinary group of RT professionals.

To our knowledge, there are no easily accessible guidelines for how to create sustainable booking scenarios or systems that automatically provide information on prospective linac utilization rates. Although historical data can be retrieved from current RT systems, e.g. InSightive Analytics (Varian Medical Systems Inc., Palo Alto, CA, USA), these data are not readily available to assist in the scheduling of RT. The booking and coordination actions presently rely on, and are left to, the staff responsible for the booking task. Different initiatives to develop methods to handle RT scheduling have been suggested but these methods are complex, and the results are rarely, if ever, implemented in reality [4], [5]. Methods that are more intuitive are needed to guide the staff when scheduling upcoming patients for treatment and how to balance available resources with short- and long-term demands. Knowledge about the current use of available linacs is important, both with respect to the time available for treatments and the time taken by other, non-treatment events. With increased understanding of department specific ratios between scheduled levels and maximum capacity, measures and priorities can be made in advance (prospective planning) to smoothly handle, or even avoid, unwanted scenarios.

The aim of this work was to use real clinical data to identify temporal trends in the scheduling of patients for external beam RT. To investigate this, we collected planned and observed (actual) scheduling data from an eight-linac modern RT department for each weekday during a two-year study period in 2018–2020. As a practical example on how to use the concept, a secondary aim was to develop a clinically useful tool to enable an overview of future scheduling levels to aid the staff in booking the desired level of new patients starting treatment per specified time period.

## Material and methods

Scheduling data for linacs were collected at the Sahlgrenska University Hospital in Gothenburg, Sweden, between February 26, 2018, and January 28, 2020, from the ARIA Oncology Information System (OIS, ver. 13.6 and 15.5; Varian Medical Systems). This hospital has a catchment area covering almost 20% of the Swedish population. The RT department is located at two sites, Gothenburg and Borås. This study examined data from the Gothenburg site only. At this site approximately 3,000 patients are treated each year. All types of cancers are treated, both with palliative and curative intent (approximately two-thirds are treated with curative intent). Special treatments such as total body irradiation and pediatric treatments, sometimes including anesthesia, are offered. There are approximately 100 healthcare professionals working at the department. Scheduling of treatments is done by nurses/administrative staff, who prioritize patients according to local guidelines. Certain diagnoses have since 2015 pre-booked slots for preparations and treatment to assure adherence to waiting time limits according to Swedish National Guidelines for cancer care [6] (*cf.* Danish *pakkeforløb* [7]; waiting time limits and RT treatment recommendations for the main cancer sites are available in Supplementary Table 1).

### The clinic

The Gothenburg site consists of eight linacs, four Clinac iX, two TrueBeam, and two TrueBeam STx machines (Varian Medical Systems). Patients can easily be transferred within these three groups of linacs. All linacs can provide image-guided RT (IGRT; mainly by daily orthogonal kV-imaging but also cone-beam CT) as well as volumetric-modulated arc therapy (VMAT).

Opening hours of the studied RT department are 6.45/7.00 AM to 4.15 PM Mondays to Fridays, allowing activities to be scheduled for at most 47 h and 30 min per linac and week (including linac quality assurance [QA] every morning and other tasks not exclusively involving beam on-time for patient treatments). Regular linac QA (monthly) typically starts at 3 PM, however, many QA activities are scheduled outside the department opening hours and have not been investigated in this study. A typical treatment slot is 15–20 min but depends on the complexity of treatment technique and is adjusted to meet individual needs. Number of fractions per treatment series for this kind of department in Sweden have previously been reported to an average of 12.2 [8].

### Scheduling data

Data on scheduled time slots for all linacs during the abovementioned period were extracted from ARIA. A dedicated database query was run daily at 6 AM to collect information about planned appointments for the present day (Day 0) and for the forthcoming 100 weekdays (20 weeks; Monday-Friday). This planning horizon was identified in a pilot-study to capture all treatment scheduling events at the investigated department. Observed scheduling data were also extracted for this same day at 9 PM (after daily work finished), to capture over-the-day deviations and differences from the planned morning schedule.

Time slots were assessed in hours and sorted into two main categories, *Treatment* and *Non-Treatment* and into nine subcategories, details in Table 1. In addition, time slots identified as patient no-shows were also collected.

**Table 1:** Scheduling categories.

Category	Description
**Treatment**	**Linac dedicated to patients**
Pre-booked	Placeholder for patients following standardized care paths
Treatment	Patient treatment including undress/dress, positioning, image verification, beam delivery
**Non-Treatment**	**Linac not available for patients**
Break	Staff breaks
Education	Education, seminars, etc.
Linac QA	Quality assurance on the linac or supporting devices
Maintenance	Maintenance or equipment downtime
Meetings	Staff meetings
Patient QA	Quality assurance of patients' treatment plans
Preparation	Time for staff to prepare for next patient (typically for stereotactic treatments)

### Analysis

Scheduling data were averaged over the whole machine park, as well as analyzed for each linac separately. In addition, three treatment-equivalent groups of linacs were analyzed separately (Group 1 [Clinac iX]: Linac 1–4; Group 2 [TrueBeam]: Linac 5–6; Group 3 [TrueBeam STx]: Linac 7–8). Linac utilization rate (LUR) was calculated by dividing the time of scheduled slots (assuming they reflected the actual time needed for different events) with opening hours, for details and variables used, see Table 2. Scheduling data were analyzed for how frequently the LUR changed between planned and observed levels for Day 0 and for how planned scheduling levels, prospective LUR, changed with different planning horizons, both with respect to treatment events (LUR_T_) and non-treatment events (LUR_NT_). The prospective LUR was fitted to a polynomial curve; the planning horizon for when LUR had largest increase per day was identified using the second order derivative of the polynomial curve fit. Comparisons of planned and observed median values were performed using the Wilcoxon signed-rank test, with two-sided p-values ≤ 0.05 being considered statistically significant. Normally distributed data are given as mean and standard deviation and non-normally distributed data are presented as median and range, unless otherwise stated. Calculations and data handling were conducted in Excel (ver. 2016; Microsoft Corporation, Redmond, WA, USA) and MATLAB (ver. R2019b; The MathWorks Inc., Natick, MA, USA).

**Table 2:** Definition of the variables used for calculations of linac utility rate (LUR).

**Variable name**	**Definition**
*Opening hours***(OH_1_)**	Linac opening hours*
*Scheduled hours outside opening hours***(OH_2_)**	Time for activities scheduled outside opening
*Hours for treatment***(HT)**	Time dedicated to patients' treatments during OH_1_ and OH_2_, no-shows excluded
*Hours for non-treatment activities within opening hours***(HNT)**	Time for non-treatment activities within opening hours
*Linac utility rate* for treatment **(LUR_T_)**	Share of treatments during a day given in percent and calculated as LUR_T_=(HT/OH_1_)*100
*Linac utility rate* for non-treatment **(LUR_NT_)**	Share of non-treatments during a day given in percent and calculated as LUR_NT_=(HNT/OH_1_)*100
*Total Linac utility rate***(LUR)**	Calculated as LUR = LUR_T_ + LUR_NT_

* Total opening time for all linacs: 375 h per week, linacs 1–4 open 7:00 AM to 4:15 PM (9.25 h per day, 46.25 h per week) and linacs 5 to 8, 6:45 AM to 4:15 PM Monday to Friday (9.5 h per day, 47.5 h per week).

### Tool development and evaluation

As a clinical example of how LUR can be applied in the clinic, a tool utilizing the concept of LUR was developed for identification of current and future scheduling levels. The tool was created in Microsoft Excel using Visual Basic for Applications (VBA). Site-specific SQL-queries was used to retrieve data from the OIS. Information retrieved was on scheduled treatment slots, prebooked treatment slots and other (non-treatment related) slots during opening hours for the forthcoming 100 weekdays. Extracted data also included the preferred date of patients first fraction and expected number of fractions for the not yet scheduled patients, as entered in the preliminary prescription by oncologists upon referral. Database retrievals and automatic calculations were designed to be triggered either by demand or on a regular basis (i.e. twice an hour). The graphical user interface was designed with three different views of information, daily (overview of scheduled hours), weekly (number of new patients scheduled to start treatment per week) and waiting patients (not yet scheduled patients and their specified starting period in weeks).

The tool was tested by four managers and one staff from the abovementioned RT department during five workshops in 2021 (April-June), they also had access to the tool between the workshops. In addition, five staff working with scheduling evaluated the tool for four weeks during the latter part of this period after initial demonstrations. To verify the usefulness of the tool, user feedback was collected in a web-based survey, including eight questions regarding user experience and future potentials of the tool (details in supplementary data). The overall impression of the tool was rated on a scale between 0 and 10 (0 = *Not good at all* and 10 = *Very good*) and future potential was reported on a four-point Likert scale (answering categories: not at all, partly agree, largely agree, completely agree). Open questions on positive and negative aspects about the tool was also included.

## Results

Due to a failure in data retrieval during an upgrade of the OIS (December 7, 2018), data for 46 weekdays were lost leaving an average of 424 weekdays per linac left for analysis. For studying different planning horizons, 37,000 prospective days per linac were used. During the studied period, one linac (linac 1) was installed and started to treat patients and one linac (linac 3) was not operated regularly due to staff shortage.

As shown in Table 3, the total median LUR for Day 0 was 87% and 89% for planned and observed, respectively (p < 0.01). The median LUR_T_ was 77%/75% for planned/observed (p < 0.01) and the LUR_NT_ was 8%/11% for planned/observed (p < 0.01). The median planned LUR_T_ for all linacs was 25%–95% and for LUR_NT_ 6%–9%. Overall, the LUR_T_ decreased between planned and observed levels while the LUR_NT_ increased. When excluding the non-fully operating lincas, Linac 1 and 3, the corresponding level for total LUR was 98%/99% for planned/observe (p < 0.01), LUR_T_ 87%/84% for planned/observed (p < 0.01) and the LUR_NT_ 8%/11% for planned/observed (p < 0.01). Time slots related to patient no-shows was in median 2.2 (range 0–8.3) hours per week for the whole machine park (0.6% of the total opening hours).

**Table 3:** Planned (start of day) and observed rates (end of day) of linac utility rate, LUR (%) for a two-year period at the Sahlgrenska University Hospital in Sweden 2018–2020.

Scheduled category	Mean ± SD [%]		Median (min─max) [%]		p-value*
	Planned	Observed	Planned	Observed	
Treatment (LUR_T_)					
Linac 1	56 ± 32	56 ± 32	63 (0─100)	63 (0─100)	<0.01
Linac 2	79 ± 26	79 ± 26	89 (0─110)	88 (0─111)	<0.01
Linac 3	31 ± 29	31 ± 29	25 (0─104)	26 (0─101)	0.31
Linac 4	82 ± 22	80 ± 23	89 (0─109)	87 (0─107)	<0.01
Linac 5	92 ± 19	91 ± 19	95 (0─122)	95 (0─127)	0.06
Linac 6	86 ± 23	85 ± 23	95 (0─127)	93 (0─122)	<0.01
Linac 7	88 ± 19	87 ± 20	93 (0─134)	92 (0─133)	<0.01
Linac 8	78 ± 27	76 ± 27	89 (0─149)	88 (0─143)	<0.01
Linac 1─4	77 ± 15	76 ± 15	77 (0─113)	76 (0─109)	<0.01
Linac 5─6	89 ± 14	88 ± 14	94 (0─112)	92 (0─110)	<0.01
Linac 7─8	83 ± 15	82 ± 15	89 (0─104)	88 (0─106)	<0.01
Total	75 ± 11	74 ± 11	77 (0─97)	75 (0─98)	<0.01

Non-treatment (LUR_NT_)					
Linac 1	11 ± 15	15 ± 19	6 (0─116)	8 (0─116)	<0.01
Linac 2	11 ± 17	14 ± 21	6 (4─116)	6 (4─137)	<0.01
Linac 3	10 ± 17	14 ± 19	6 (0─126)	6 (2─129)	<0.01
Linac 4	12 ± 19	14 ± 21	6 (0─116)	6 (0─121)	<0.01
Linac 5	10 ± 14	12 ± 15	6 (3─122)	7 (3─122)	<0.01
Linac 6	9 ± 12	11 ± 14	6 (0─122)	7 (0─122)	<0.01
Linac 7	11 ± 13	14 ± 15	8 (0─136)	9 (0─136)	<0.01
Linac 8	13 ± 17	17 ± 21	9 (0─122)	10 (0─138)	<0.01
Linac 1─4	13 ± 12	17 ± 14	8 (4─119)	12 (4─119)	<0.01
Linac 5─6	9 ± 9	12 ± 10	6 (1─65)	8 (3─66)	<0.01
Linac 7─8	12 ± 11	15 ± 13	9 (3─75)	11 (2─75)	<0.01
Total	11 ± 6	14 ± 7	8 (5─64)	11 (5─65)	<0.01

LUR_T_ + LUR_NT_					
Linac 1	68 ± 33	71 ± 34	78 (0─116)	79 (0─163)	<0.01
Linac 2	90 ± 25	92 ± 28	96 (5─189)	96 (5─190)	<0.01
Linac 3	40 ± 32	44 ± 33	35 (0─126)	39 (3─134)	<0.01
Linac 4	93 ± 17	94 ± 19	96 (0─204)	96 (0─202)	<0.01
Linac 5	101 ± 16	103 ± 16	103 (5─139)	104 (5─216)	<0.01
Linac 6	96 ± 21	96 ± 22	102 (0─134)	101 (0─166)	<0.01
Linac 7	99 ± 17	101 ± 18	102 (0─155)	103 (0─172)	<0.01
Linac 8	91 ± 24	93 ± 25	100 (0─163)	100 (0─166)	<0.01
Linac 1─4	90 ± 18	94 ± 20	90 (7─139)	94 (7─155)	<0.01
Linac 5─6	98 ± 13	100 ± 13	102 (3─132)	102 (3─135)	<0.01
Linac 7─8	95 ± 15	97 ± 16	100 (5─129)	101 (5─154)	<0.01
Total	85 ± 11	88 ± 12	87 (16─109)	89 (16─113)	<0.01

SD, Standard deviation; LUR_T_, linac utility rates for treatment events; LUR_NT_, linac utility rates for non-treatment events.

* Comparison of median values between planned and observed calculated by Wilcoxon signed-rank test. A two-sided p-value ≤ 0.05 was considered to indicate statistical significance.

### LUR changes at day 0

**Fig. 1:**
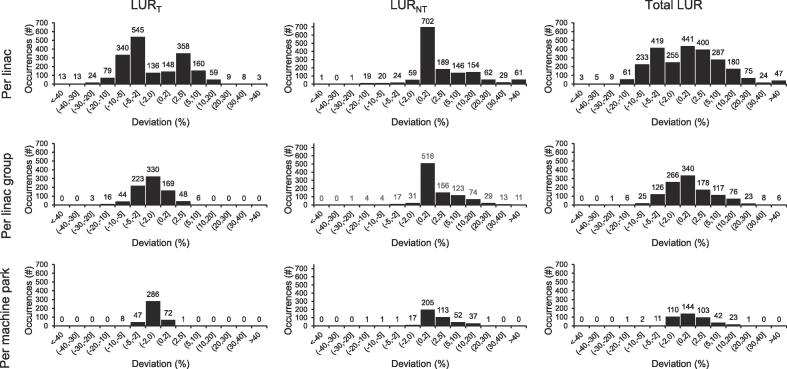
Linac utilization rate (LUR) changes per all eight linacs, the three linac groups and the whole machine park (positive/negative change is an increase/decrease in LUR between planned and observed scenarios). The histograms illustrate data at Day 0, according to scheduling categories of treatment, LUR_T_ and non-treatment, LUR_NT,_ and in total, Total LUR. Overall, 3390/1230/440 days (linacs/linac groups/machine park) were studied during the period 2018–2020.

**Fig. 2:**
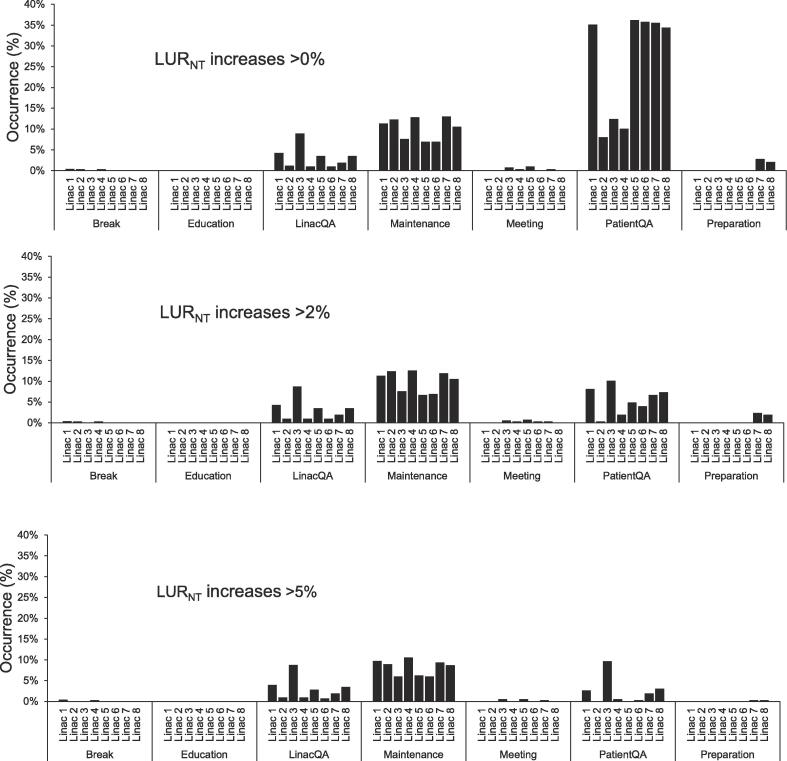
Reasons for non-treatment linac utilization rate (LUR_NT_) increases larger than 0% (top), 2% (middle) and 5% (bottom) during treatment days and their respective rate of occurrence. Data collected during a two-year period in 2018–2020, in total 3390 days were analyzed. Abbreviations: LinacQA, Quality assurance of linac; PatientQA, quality assurance of patients’ treatment plans.

Increases in LUR between planned and observed levels for any linac at Day 0 occurred 745 (22%) times for LUR_T,_ 1343 (40%) times for LUR_NT_ and 1454 (43%) times for Total LUR during the studied period (Fig. 1). Decreases for any linac occurred 1150 (34%) times for LUR_T,_ 124 (4%) times for LUR_NT_ and 985 (29%) times for Total LUR. Deviations (increases or decreases) from planned levels occurred 89% of the 3390 investigated linac days, at group and department levels the schedule deviated 74% and 99% of all days, respectively. The most common reason for small increases (≤2%) in LUR_NT_ was patient QA and for larger increases (>5%) maintenance (Fig. 2). Linac 3 had similar degree of patient QA regardless of increase cut off.

### Prospective LUR

**Fig. 3:**
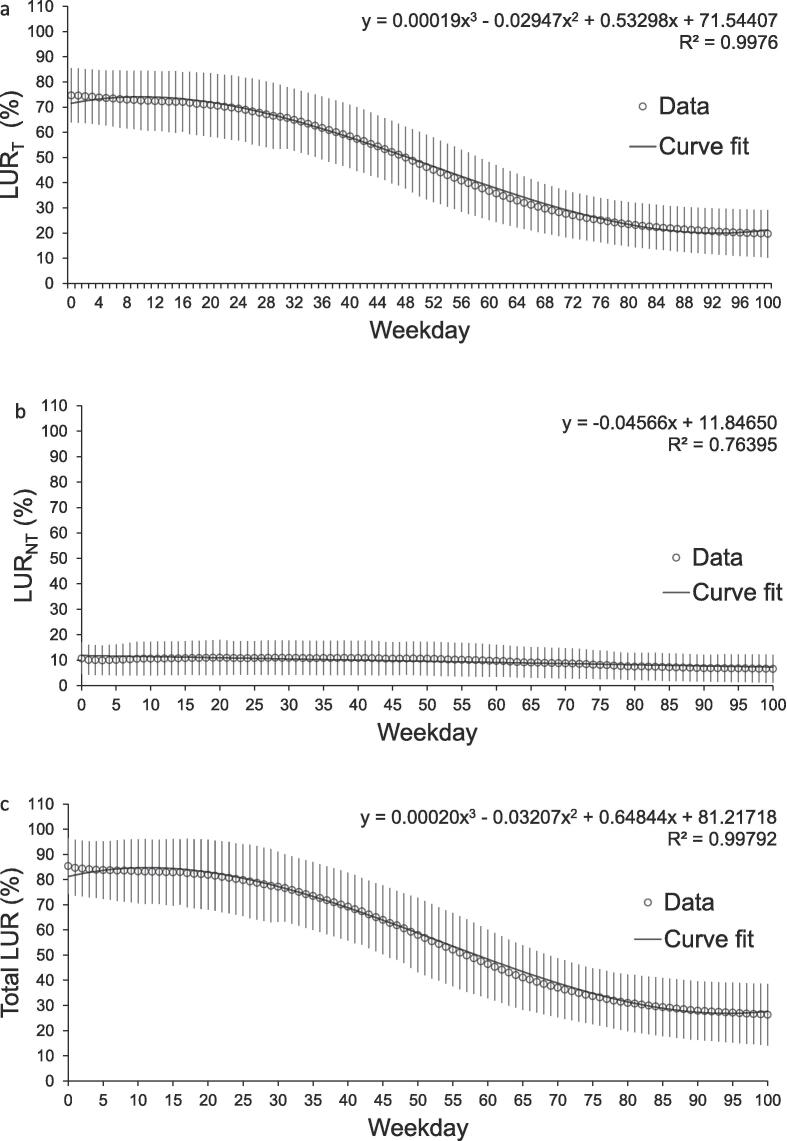
Planned linac utilization rate (LUR) for present (Day 0) and forthcoming 100 weekdays (20 weeks) for the whole machine park, showing the mean values of all collections during the studied two-year period in 2018–2020. Data are shown for scheduling categories: treatment, LUR_T_ (a), non-treatment, LUR_NT_ (b) and in total, LUR (c). Error bars represent one standard deviation.

The mean LUR_T_ gradually increased with shorter planning horizon (Fig. 3). LUR_NT_ was between 7% and 11% for the entire studied period. The largest increase in LUR_T_ for the individual linacs was between days 51 and 56 for the fully operating linacs and at day 36 and 87 for Linac 1 and Linac 3 respectively, details in supplementary Figs. 1 and 2. The highest LUR_T_ occurred at Day 16 for linac groups 1 and 2 and at Day 0 for Group 3. For the whole machine park, the maximum occurred at Day 0, when only including the fully operating linacs the maximum occurred at Day 16. The total LUR followed the same trend as LUR_T_. An analysis for a sub-period (September-November 2018) excluding the Swedish summer vacation period showed higher LUR_T_ (maximum: 75% compared to 78%; minimum: 23% compared to 20%) and smaller standard deviation (mean SD: 5% compared to 11%) but a similar LUR_NT_ as for the complete data set, details in supplementary Fig. 3. The mean LUR of pre-booked slots was 20% at day 100 and the transition from pre-booked to scheduled treatments started around weekday 45 and reached 1% at day 10 (data no shown). Prospective LUR was also analyzed using median values, showing minor deviations from mean but with no impact on overall conclusions (data not shown).

### Tool for clinical use

The tool has three main views: daily, weekly and waiting patients. The daily view presents an overview of scheduled hours for individual linacs and the mean level for treatment-equivalent groups of linacs as well as over the whole machine park (supplementary Figure 4). The weekly view shows total number of new patients scheduled to start treatment per week and for the two largest diagnosis groups, at the studied RT department (supplementary Figure 5). The view of waiting patients shows the number of not yet scheduled patients and their specified starting period in weeks (supplementary Figure 6).

The tool was introduced at the RT department in April 2021 and user feedback was collected from 3/4 managers (75%) and 5/6 staff (83%) after a testing period of twelve and four weeks, respectively. Total rating of the tool was 7 ± 2 (mean ± SD). All but one (7/8) responded positive for potential of the tool to 1) ease their work as well as 2) contribute to a more even scheduling. Three of five staff reported the tool to include too much information in its current version.

## Discussion

In this study, we investigated scheduling data for an eight-linac modern RT department in Sweden. Using real-world information from the oncological information system during two years, we found that about three quarters of the opening time was dedicated to treating patients. Increases from the planned schedule at any linac occurred almost every second day and major unforeseen events were mainly related to non-treatment events, for which the main reason was maintenance/downtime. Patients not showing up for treatments were rare. The prospective utilization rates showed that the non-treatment level was stable over time while the treatment level increased rapidly about ten weeks before any given date. The total utilization rate reached its maximum for the fully-operating linacs already between two to four weeks ahead of this same date. We also demonstrated how the concept of linac utilization rate could be practically used as a scheduling aid for RT managers and staff to provide an overview of current and planned scheduling levels.

Our data showed that most treatment slots were filled weeks in advance and left little room for patients in need of acute treatment or for handling unforeseen events. When scheduling patients a long time in advance, cancellations or timing of other treatments (i.e., surgery and chemotherapy) may cause several cases of rescheduling. It is also problematic to schedule a new patient to a highly utilized linac since it requires available slots for a complete treatment series. Our data on frequently decreased treatment levels within a day also quantify the reoccurring need for rescheduling. Postponed treatments must be compensated to not jeopardize intended biologic effects [9], [10], but by adding extra treatments future utilization rates will increase and reduce the number of available treatment slots. This means that ongoing treatments need to be prioritized over those patients who have not yet started RT if the capacity is limited, which also can result in increased waiting times to treatment for the latter. Thomsen et al. have used a model for managing capacity to improve transparency in the booking process and to derive easily prospective waiting times and utilization rates [5]. They used the average amount of appointments and new starts per day in combination with waiting time limits to set upper and lower limits for their scheduling. But they assumed the same slot time for all patients, which may be problematic for patients who require deviating (long) slot times. We analyzed planned and observed scheduling levels of treatment and non-treatment events and identified specific time points where the overall scheduling scenario changed rapidly. There seems to be one easy bulk-booking period which fills the majority of treatment slots weeks ahead of a given date and one difficult period of handling acute events with short notice using the few remaining non-booked slots. The developed tool to aid booking staff with scheduling new patients clearly presents the current number of scheduled new patients per week and the LUR per day, per linac, linac group and the whole department, illustrating the abovementioned phenomenons and increasing chances to adhere to set scheduling levels.

Strengths of our study are that we used real-world data over a long period of time for a large modern RT department with representable linac utilization levels (our data is in line with Legrain et al. who identified utilization levels for a fully-operaing linac around 80% when simulating over a planned horizon of 300 days in 2009 using data from a large RT department [11]). One weakness is that we assumed fixed opening hours for all weekdays in our calculations, which for instance, did not capture less capacity during longer vacation periods. An analysis of a three-month period outside the Swedish summer vacation period, however, showed a slightly higher utilization, indicating that the presented results somewhat underestimate utilization rates for non-vacation periods. Another drawback is that our study only captures activities that appear in the schedule, and this may not fully reflect the real situation. Regardless of a scheduling slot is too short or too long in reality, we motivate our choice with that this is what is assessed when booking staff search for empty slots for a new patient. The actual use needs to be further studied. It is also important to remember that regular QA activities scheduled to start within the opening hours affects LUR_NT_ at a department specific level. Even if the developed tool in its current version received overall positive feedback from the users, the evaluation was informative although small scale. The managers also received more support and could evaluate the tool longer compared to the staff, indicating that a four-week trial period may be too short to understand all information and that future improvements potentially are needed to for instance divide the tool into two parts with different levels of detail, one for strategic overview and one for daily use. Still, we believe that further development of the tool, with insights gained from this work and from the work by Thomsen [5], may be beneficial in terms of a decision-support system for the scheduling task. While our presented results are department-specific, the concept and use of prospective planning can be used by others after calibration to their data.

## Conclusion

Regular analysis of the planned scheduling level of RT in line with our presented strategy here can be useful for the identification of deviating booking scenarios and assist in sustainable planning of both short- and long-term booking scenarios, staffing, and equipment requirements.

Alterations in the linac schedule occurs frequently and often with short notice. To treat all patients according to plan, different actions for short- and long-term planning horizons are required. For patients undergoing treatment, strategies such as having extra spare capacity to complete all treatments during opening hours (schedule fewer patients per day) or assuring that staff is prepared to work beyond opening hours each day becomes critical. Careful scheduling will be even more important when the use of online adaptive techniques increases. Even if not realistic scenarios for many RT departments, having a linac on standby seems beneficial, both for patient QA and, as back-up for unforeseen events and equipment downtime. A more realistic scenario are groups of equal linacs which are helpful when a patients’ primary linac is down for maintenance. A tool that aggregates information on scheduled activities and presents informative views about current and future scheduling levels can aid RT staff to achieve a more even workload throughout the RT process than what is possible using current strategies.

## Declaration of Competing Interest

The authors declare that they have no known competing financial interests or personal relationships that could have appeared to influence the work reported in this paper.
