# Supplementary material for: Linear accelerator utilization: Concept and tool to aid the scheduling of patients for radiotherapy
Source: Tech Innov Patient Support Radiat Oncol. 2021 Sep 30;20:10–6. doi: 10.1016/j.tipsro.2021.09.001 (PMC8531843; doi:10.1016/j.tipsro.2021.09.001)
Supplement: Supplementary data 1 [file mmc1.pdf]

Questionnaire about the tool for booking overview

1. What is your role?

- ◯ Employee
- ◯ Manager

2. My overall assement of the booking overview

0	1	2	3	4	5	6	7	8	9	10

Not good at all — Very good

3. It was easy to use the booking overview

0	1	2	3	4	5	6	7	8	9	10

Not at all — Very easy

4. The booking overview has potential to

	Not at all	Partly agree	Largely agree	Completely agree
Ease my work	◯	◯	◯	◯
Contribute to a more even scheduling of patients	◯	◯	◯	◯
Contribute to a more even distribution of starting patients	◯	◯	◯	◯
Be a tool for regular use	◯	◯	◯	◯

5. I thought this was particulary good with the booking overview

6. This should be improved in the next version of the tool for booking overview
