# Supplementary material for: Linear accelerator utilization: Concept and tool to aid the scheduling of patients for radiotherapy
Source: Tech Innov Patient Support Radiat Oncol. 2021 Sep 30;20:10–6. doi: 10.1016/j.tipsro.2021.09.001 (PMC8531843; doi:10.1016/j.tipsro.2021.09.001)
Supplement: Supplementary data 2 [file mmc2.pdf]

Supplementary material

Supplementary Table 1. Waiting time limits and RT treatment recommendation for the main cancer sites according to Swedish national guidelines (*Kunskapsbank för cancervården* [Eng*: Knowledge bank for cancer care*]. https://kunskapsbanken.cancercentrum.se/diagnoser/ Accessed: 2021-09-23).

**Cancer type**	**Time from well-founded suspicion of cancer to start of treatment (days)**	**Time from cancer diagnosis to first treatment (days)**	**RT**
Breast	28	14 (surgery)<br>42 (from surgery to adjuvant RT)	40 Gy / 15 frac.<br>Additional boost of 10-16 Gy (age ≤ 50yr or non-radical surgery)
Prostate	75 (RT)<br>57 (RT and hormones)	21 (RT)<br>3 (hormones)	42.7 Gy / 7 frac. or 60 Gy 20 frac. (Intermediate risk)<br>78 Gy / 39 frac. (high or very high risk)
NSCL	44	14 (surgery or RT)	45 Gy / 3 frac. (Stage II)<br>66-68 Gy / 33-34 frac. (Stage III)
Rectal	39	14 (surgery or RT)	5 Gy / 5 frac. (neoadjuvant or preoperative)

Abbreviations: frac=fraction, Gy=Gray, NSCL=non-small-cell lung (cancer), RT=radiotherapy, yr=year.

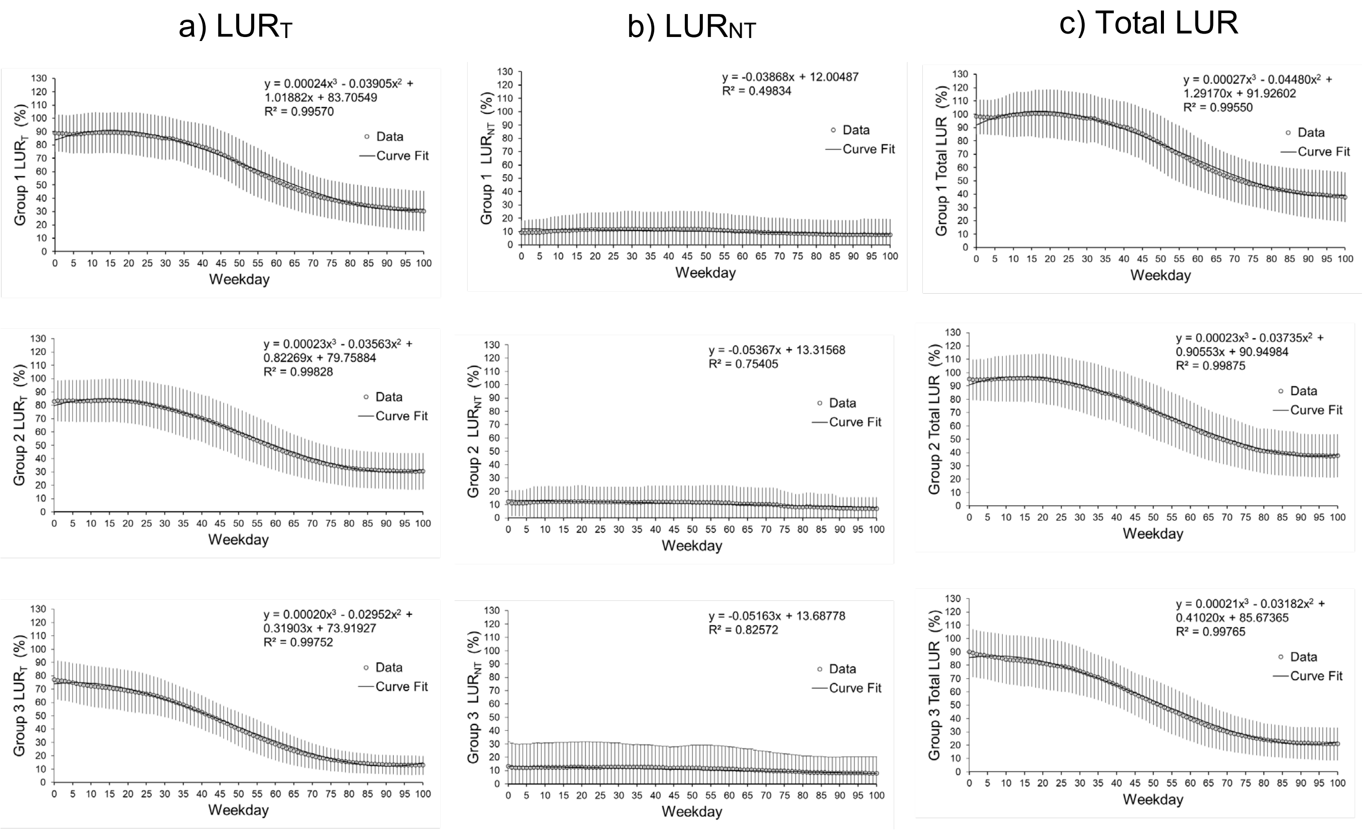

Supplementary Figure 1. Planned linac utility rate (LUR) for present (Day 0) and forthcoming 100 weekdays (20 weeks) for the three linac groups, Group 1 (linac 1-4 [Clinac iX]; top), Group 2 (linac 5-6 [Truebeam]; middle), Group 3 (linac 7-8 [Truebeam STx]; bottom), showing the mean values of all collections during the studied two-year period in 2018–2020. Data are shown for scheduling categories: treatment, LUR_T (a), non-treatment, LUR_{NT} (b) and in total, LUR (c). Error bars represent one standard deviation.

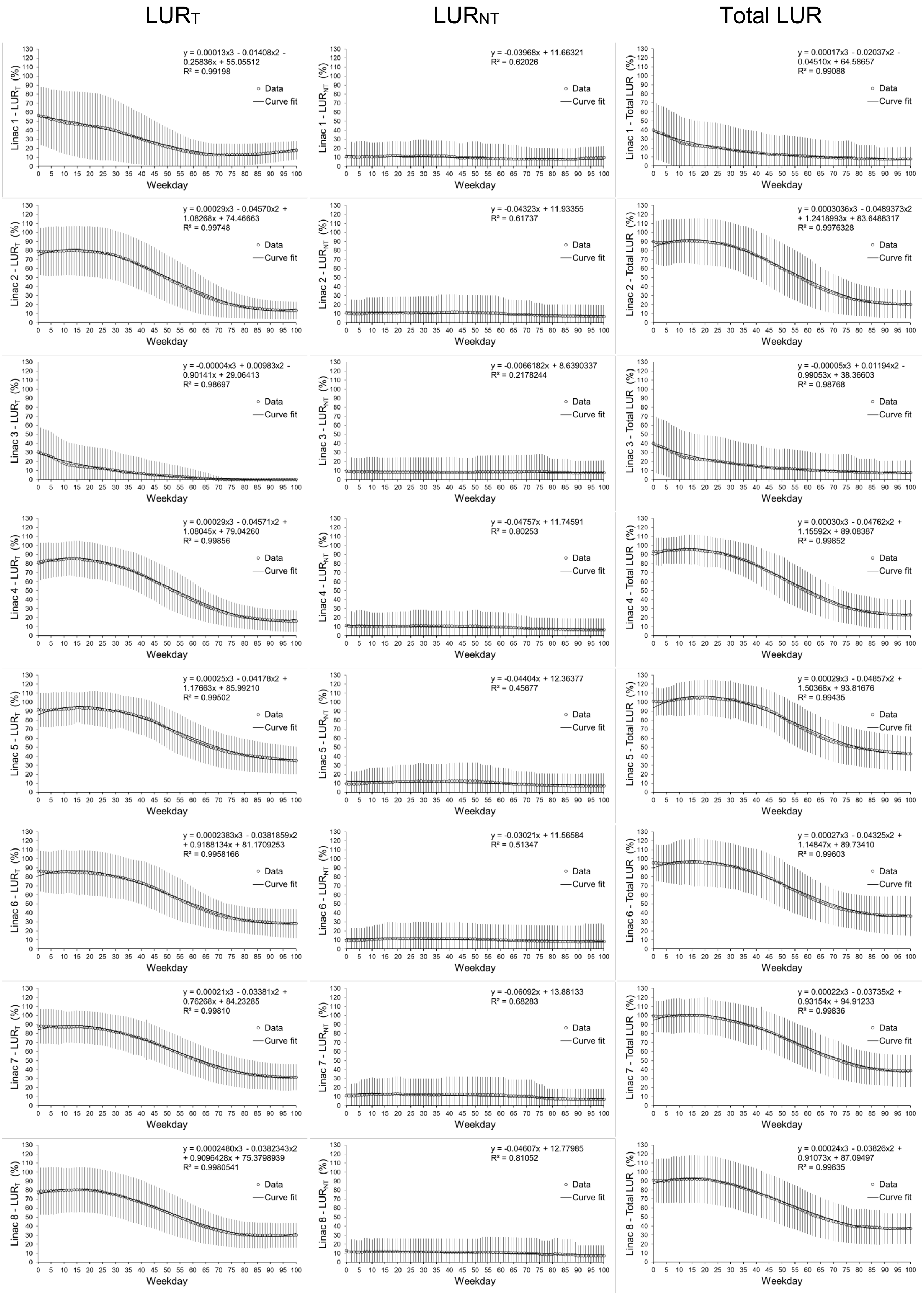
LURT
LURNT
Total LUR
y = 0.00013x3 - 0.01408x2 - 0.25836x + 55.05512
R² = 0.99198
y = -0.03968x + 11.66321
R² = 0.62026
y = 0.00017x3 - 0.02037x2 - 0.04510x + 64.58657
R² = 0.99088
y = 0.00029x3 - 0.04570x2 + 1.08268x + 74.46663
R² = 0.99748
y = -0.04323x + 11.93355
R² = 0.61737
y = 0.0003036x3 - 0.0489373x2 + 1.2418993x + 83.6488317
R² = 0.9976328
y = -0.00004x3 + 0.00983x2 - 0.90141x + 29.06413
R² = 0.98697
y = -0.0066182x + 8.6390337
R² = 0.2178244
y = -0.00005x3 + 0.01194x2 - 0.99053x + 38.36603
R² = 0.98768
y = 0.00029x3 - 0.04571x2 + 1.08045x + 79.04260
R² = 0.99856
y = -0.04757x + 11.74591
R² = 0.80253
y = 0.00030x3 - 0.04762x2 + 1.15592x + 89.08387
R² = 0.99852
y = 0.00025x3 - 0.04178x2 + 1.17663x + 85.99210
R² = 0.99502
y = -0.04404x + 12.36377
R² = 0.45677
y = 0.00029x3 - 0.04857x2 + 1.50368x + 93.81676
R² = 0.99435
y = 0.0002383x3 - 0.0381859x2 + 0.9188134x + 81.1709253
R² = 0.9958166
y = -0.03021x + 11.56584
R² = 0.51347
y = 0.00027x3 - 0.04325x2 + 1.14847x + 89.73410
R² = 0.99603
y = 0.00021x3 - 0.03381x2 + 0.76268x + 84.23285
R² = 0.99810
y = -0.06092x + 13.88133
R² = 0.68283
y = 0.00022x3 - 0.03735x2 + 0.93154x + 94.91233
R² = 0.99836
y = 0.0002480x3 - 0.0382343x2 + 0.9096428x + 75.3798939
R² = 0.9980541
y = -0.04607x + 12.77985
R² = 0.81052
y = 0.00024x3 - 0.03826x2 + 0.91073x + 87.09497
R² = 0.99835
Data
Curve fit
Weekday

Supplementary Figure 2. Planned linac utility rate (LUR) for present (Day 0) and forthcoming 100 weekdays (20 weeks) for the individual eight linacs, showing the mean values of all collections during the studied two-year period in 2018–2020. Data are shown for scheduling categories: treatment, LUR_T (left), non-treatment, LUR_{NT} (middle) and in total, LUR (right). Error bars represent one standard deviation.

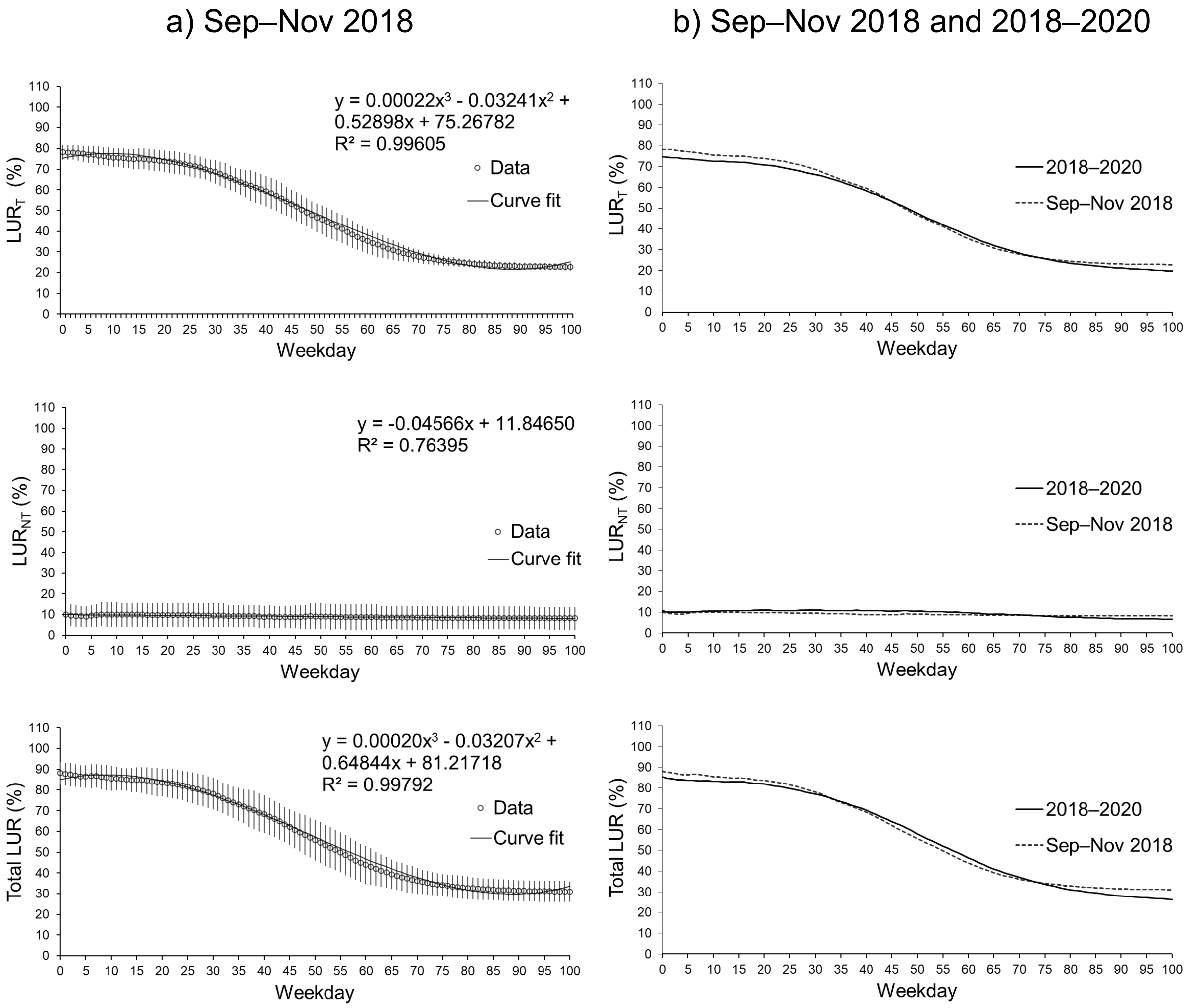

Supplementary Figure 3. Planned linac utility rate (LUR) for present (Day 0) and forthcoming 100 weekdays (20 weeks) for the whole machine park, showing the mean values of all collections during the (a) sub-period September to November 2018 (not including any longer vacation periods) and (b) the sub-period together with the complete period. Data are shown for scheduling categories: treatment, LUR_T (top), non-treatment, LUR_{NT} (middle) and in total, Total LUR (bottom). Error bars represent one standard deviation.

Per day

Last updated: 2021-06-22 12:29

Opening hours (h)	08:45			
07:52	90%	< Yellow		
08:34	98%	> Green	Count mean value on	
08:55	102%	< Green > Red	4 / 2 / 2 / 8	
09:16	106%	> Dark red		

Count treatment time	Yes
Count prebooked	Yes
Count other	Yes

Extract new data from ARIA

Wee	Weekda	Date	Linac1	Linac2	Linac3	Linac4	Linac5	Linac6	Linac7	Linac8	Starts	Grp1	Grp2	Grp3	Total (i
25	monday	2021-06-21	09:15	08:10	01:45	07:30	08:15	07:50	07:45	07:35	19	06:40	08:02	07:40	83%
25	tuesday	2021-06-22	04:40	06:20	03:15	08:30	07:05	08:45	08:50	08:25	18	05:41	07:55	08:37	80%
25	wednesday	2021-06-23	04:20	08:10	04:20	06:05	08:35	08:40	08:55	07:55	22	05:43	08:37	08:25	81%
25	thursday	2021-06-24	05:25	07:50	00:00	07:00	05:10	07:50	08:20	06:20	15	05:03	06:30	07:20	68%
25	friday	2021-06-25	00:00	00:00	00:00	00:00	00:00	00:00	00:00	00:00	0	00:00	00:00	00:00	0%
25	saturday	2021-06-26	00:00	00:00	00:00	00:00	00:00	00:00	00:00	00:00	0	00:00	00:00	00:00	0%
25	sunday	2021-06-27	00:00	00:00	00:00	00:00	00:00	00:00	00:00	00:00	0	00:00	00:00	00:00	0%
26	monday	2021-06-28	00:35	08:35	00:15	00:05	08:25	08:25	08:45	06:25	10	02:22	08:25	07:35	59%
26	tuesday	2021-06-29	00:15	08:25	00:15	01:50	06:55	08:45	08:00	05:40	7	02:41	07:50	06:50	57%
26	wednesday	2021-06-30	00:15	08:25	00:15	00:00	07:55	08:45	08:10	06:45	10	02:13	08:20	07:27	58%
26	thursday	2021-07-01	00:15	08:15	00:15	00:05	06:05	08:10	08:25	06:50	8	02:12	07:07	07:37	55%
26	friday	2021-07-02	00:15	08:00	00:15	00:05	07:50	07:15	06:20	06:30	4	02:08	07:32	06:25	52%
26	saturday	2021-07-03	00:00	00:00	00:00	00:00	00:00	00:00	00:00	00:00	0	00:00	00:00	00:00	0%
26	sunday	2021-07-04	00:00	00:00	00:00	00:00	00:00	00:00	00:00	00:00	0	00:00	00:00	00:00	0%
27	monday	2021-07-05	00:15	08:30	00:15	00:05	07:50	08:00	08:05	07:40	16	02:16	07:55	07:52	58%
27	tuesday	2021-07-06	00:15	09:20	00:15	00:55	06:50	06:55	13:05	06:55	6	02:41	06:52	10:00	64%
27	wednesday	2021-07-07	00:15	08:25	00:15	00:05	07:50	08:05	13:55	08:30	9	02:15	07:57	11:12	68%
27	thursday	2021-07-08	00:15	08:05	00:15	00:05	07:40	08:50	08:00	15:25	12	02:10	08:15	11:42	69%
27	friday	2021-07-09	00:15	06:55	00:15	00:05	09:00	07:45	06:15	14:55	3	01:52	08:22	10:35	65%
27	saturday	2021-07-10	00:00	00:00	00:00	00:00	00:00	00:00	00:00	00:00	0	00:00	00:00	00:00	0%
27	sunday	2021-07-11	00:00	00:00	00:00	00:00	00:00	00:00	00:00	00:00	0	00:00	00:00	00:00	0%
28	monday	2021-07-12	00:15	08:45	00:15	00:05	09:00	08:35	08:45	04:30	15	02:20	08:47	06:37	57%
28	tuesday	2021-07-13	00:15	09:50	00:15	00:05	07:10	08:10	07:45	04:50	5	02:36	07:40	06:17	55%
28	wednesday	2021-07-14	00:15	08:55	00:15	00:05	08:40	09:00	05:40	06:10	5	02:22	08:50	05:55	56%
28	thursday	2021-07-15	00:15	08:35	00:15	00:05	08:40	09:20	08:35	03:50	8	02:17	09:00	06:12	57%
28	friday	2021-07-16	00:15	08:20	00:15	00:05	09:25	08:25	07:55	03:00	5	02:13	08:55	05:27	54%
28	saturday	2021-07-17	00:00	00:00	00:00	00:00	00:00	00:00	00:00	00:00	0	00:00	00:00	00:00	0%
28	sunday	2021-07-18	00:00	00:00	00:00	00:00	00:00	00:00	00:00	00:00	0	00:00	00:00	00:00	0%

Supplementary Figure 4. Screenshot of the developed scheduling tool with future scheduling levels per day for all linacs (example data), linacs groups and the overall department level. Information is dynamically color-coded based on user defined LUR thresholds with two levels of yellow illustrating that there is room for additional patients (light/dark yellow=a lot/little room), green illustrating fully booked, and two levels of red illustrating overbooked (light/dark red=little/much overbooked). The three grey buttons (top left) enable the user to choose which scheduling categories to include in the calculations. The two framed boxes (top right) allow the user to relate various opening hours to the color coding and also to adjust how many linacs in each group to use in calculations of the mean value, for instance if any of the linacs should be excluded because of longer periods of downtime etc.

Last updated: 2021-06-22 12:29

Update starts per week

☐ Auto update (every 30e min)

< Light yellow	5	5	5	5	75
< Yellow = Green > Red	**14**	**8**	**18**	**9**	**95**
> Dark red	17	12	23	13	105

a

Starts per week

Week	Prostate	Mam coordin	Mam others	All diagnos	Pre booked not used
25	12	7	0	74	0
26	3	14	1	39	0
27	5	13	0	46	8
28	5	7	0	38	16
29	5	5	0	38	15
30	5	6	0	34	17
31	2	13	7	42	19
32	1	10	1	39	21
33	3	10	7	42	21
34	10	4	20	60	23
35	5	5	10	41	21
36	2	1	1	28	23
37	0	4	2	28	22
38	0	3	1	27	23
39	0	3	0	25	22
40	0	0	0	22	22
41	0	0	0	21	21
42	0	0	0	22	22

b

Linac1

Week	Prostate	Mam coordi	Mam othe	All diagno	Pre booked not used
25	0	1	0	6	0
26	0	2	0	3	0
27	0	1	0	5	1
28	0	2	0	7	3
29	0	0	0	5	4
30	0	0	0	4	4
31	0	3	6	13	4
32	0	3	1	7	3
33	0	2	3	13	6
34	0	0	6	11	3
35	0	2	1	7	4
36	0	0	0	4	4
37	0	2	0	6	4
38	0	2	0	6	4
39	0	1	0	5	4
40	0	0	0	4	4
41	0	0	0	4	4
42	0	0	0	4	4

c

Starts per lianc (all diagnoses)

Color codig: White (few starts) to blue (highest amout of starts)

Vecka	Linac1	Linac2	Linac3	Linac4	Linac5	Linac6	Linac7	Linac8
25	6	4	4	7	7	9	9	9
26	3	0	3	7	0	6	8	7
27	5	0	8	12	0	9	4	7
28	7	0	7	10	0	6	5	0
29	5	0	6	9	0	5	9	0
30	4	0	4	6	0	5	7	0
31	13	0	3	5	0	3	6	0
32	7	0	3	3	0	6	4	7
33	13	0	3	3	0	3	5	8
34	11	7	2	5	7	5	6	7
35	7	4	2	3	4	4	4	2
36	4	1	2	2	4	2	3	2
37	6	1	2	3	2	1	4	3
38	6	1	2	2	3	1	3	3
39	5	1	2	3	2	2	2	3
40	4	1	2	2	2	2	2	2
41	4	1	2	2	2	2	2	1
42	4	1	2	2	2	1	3	2

Supplementary Figure 5. Weekly overview (example data) of number of new patients starting treatment and the investigated department's choice of diagnoses (prostate, breast cancer in need for coordination of other treatments, e.g. chemotherapy and other breast cancer treatments) and the overall total, color-coded similar to Supplementary Figure 4 (a). Details on number of patients waiting to be scheduled and their respective preferred starting period (b). Overview of number of starting patients per week per linac. Color-coded white to blue relative to the maximum number of starting patients (white=lowest number patients, dark blue=highest number of patients) (c).

a

Activity	Open	In Progress	Total
Book Urgent	0	0	0
Book single fraction	3	0	3
Book Gold markers	25	8	33
Book Mamillary	1	0	1
Book Palliative	36	0	36
Book Post-op	8	2	10
Book Coordination	53	30	83
Book Specials	5	2	7
Book other	51	16	67
Total	182	58	**240**

b

Week	Starting week according to oncologists assessment		
	Preferred	Latest	Latest & In progress
Earlier		1	0
25	20	6	3
26	40	8	1
27	32	16	6
28	12	37	4
29	13	25	1
30	15	15	6
31	10	17	3
32	13	11	1
33	12	20	9
34	5	18	6
35	13	17	2
36	9	10	4
37	8	9	4
38	5	9	3
39	4	8	1
40	2	3	2
41	2	3	0
42	3	2	0

c

Fractions to shedule	Number	Time (min)	Total time (h)
1st fraction	240	30	120
Remaining	2792	15	698
Total	3032		818

Supplementary Figure 6. Overview of waiting not yet scheduled patients (example data) and their associated category (a). Weekly overview of patients starting period according to an oncologist's assessment of the referral (b). Total number of unscheduled fractions according to the initial assessment (c).
